# Osteoporotic fractures and subsequent fractures: imminent fracture risk from an analysis of German real-world claims data

**DOI:** 10.1007/s00404-021-06123-6

**Published:** 2021-07-11

**Authors:** Peyman Hadji, Bernd Schweikert, Edda Kloppmann, Patrick Gille, Lars Joeres, Emese Toth, Luis Möckel, Claus-C. Glüer

**Affiliations:** 1 Frankfurt Center of Bone Health, Philipps-University of Marburg, Frankfurt, Germany; 2 ICON RWESA, Munich, Germany; 3 Vilua Healthcare GmbH, Munich, Germany; 4 UCB Pharma, Monheim, Germany; 5 UCB Pharma, Brussels, Belgium; 6 HSD Hochschule Döpfer GmbH, University of Applied Sciences, Cologne, Germany; 7 Biomedical Imaging Section, Department of Radiology and Neuroradiology, Universitätskrankenhaus Schleswig-Holstein, Christian-Albrechts-Universität zu Kiel, Kiel, Germany; 8 Department of Radiology and Neuroradiology, Molecular Imaging North Competence Center, Am Botanischen Garten 14, 24118 Kiel, Germany

**Keywords:** Fragility fracture, Fracture risk assessment, German population, Imminent risk, Osteoporosis, Real-world evidence

## Abstract

**Purpose:**

In osteoporosis, prior fracture is a strong predictor of subsequent fracture. This study aimed to assess the imminent risk of subsequent fracture following an initial fracture in osteoporosis patients in Germany, and to identify clinical and demographic characteristics that are independently associated with subsequent fracture risk.

**Methods:**

In this retrospective, observational cohort study using German real-world claims data, male and female patients aged ≥ 50 years with osteoporosis who experienced an initial (“index”) hip/femur, vertebral, forearm/wrist/hand or shoulder/upper arm fracture between 2010 and 2014 were included. The incidence and timing of subsequent fractures during a 1-year follow-up period were analyzed. Independent risk factors for subsequent fracture were identified by multivariate regression analysis.

**Results:**

A total of 18,354 patients (mean age: 77 years; standard deviation: 9.8) were included. Of these, 2918 (15.9%) suffered a subsequent fracture during the 1-year follow-up period. The incidence of subsequent fracture was higher following an index vertebral fracture (18.0%) than after an index forearm/wrist/hand fracture (14.1%) or index hip/femur fracture (12.1%). Subsequent 1-year fracture incidence was generally higher in older patients. Index fracture type, age, epilepsy/use of antiepileptics, and heart failure were all independently associated with subsequent fracture risk.

**Conclusion:**

Osteoporosis patients in Germany are at imminent risk of subsequent fracture during the first year following an initial fracture. They should be targeted for immediate post-fracture treatment to reduce the risk of further fractures, especially in the presence of specific risk factors such as old age or index vertebral fracture.

**Supplementary Information:**

The online version contains supplementary material available at 10.1007/s00404-021-06123-6.

## Introduction

Osteoporosis is a progressive, systemic, skeletal disorder characterized by low bone mass, an increase in bone fragility, and susceptibility to fracture. In 2010, 22 million women and 5.5 million men were affected by osteoporosis in the European Union (EU) [1]. In Germany specifically, the estimated prevalence of osteoporosis (based on ICD-10-GM [International Classification of Diseases, 10th revision, German Modification] code M80.*/M81.*) was 4.4% in 2016, affecting approximately 3.63 million patients, 83% of whom were female [2, 3].

Osteoporosis patients are vulnerable to fragility fractures, defined as fractures that result from mechanical forces that would not normally cause a fracture, such as a fall from a standing height [4]. Fragility fractures most often affect the vertebrae of the spine, the hip/femur, and the distal radius near the wrist [4]. It is estimated that ~ 334,000 osteoporosis patients in Germany suffered new vertebral or hip/femur fractures in 2016 [3]. Fragility fractures are associated with chronic pain, disability, reduced quality of life and increased mortality [1, 5–9]. In Germany, they accounted for approximately €11 billion in healthcare-related costs and a loss of over 300,000 quality-adjusted life years in 2017 [10]. As the average age of Germany’s population increases, the incidence of fragility fractures is predicted to rise 18.5% between 2017 and 2030, while the associated annual costs are forecasted to increase by 23.2% [10]. Over the same period, the related quality-adjusted life year losses are expected to rise by 22.4% [10].

Patients who sustain an initial osteoporotic fracture are at increased risk of subsequent fractures [10–13]. In women aged 50–90 years, the risk of sustaining a fracture within a year of an initial fracture is roughly five times greater than in individuals with no prior fracture [14]. After the first year, fracture risk declines, but does not return to pre-fracture levels, remaining higher than in the general population for at least a decade [14–16]. Overall, 10–18% of individuals suffer a subsequent fracture within the 1–2 years following a prior fracture, which is known as the period of imminent risk [11, 14, 17–19]. Imminent fracture risk is particularly high in older patients and those with index vertebral fractures [20]. Despite this, many patients in Germany fail to receive osteoporosis treatment in the immediate aftermath of a fracture [10]. By minimizing this treatment gap, particularly in the subpopulations at greatest risk, the social and economic burden of osteoporosis could be reduced [20].

This study aimed to: (a) assess the imminent risk of subsequent fracture in osteoporosis patients in Germany that experience an initial (“index”) fracture; (b) characterize the incidence of different fracture types; and (c) identify clinical and demographic variables that are independently associated with subsequent fracture risk. We hypothesized that risk of fracture would be associated with the incidence of a recent fracture, amongst other factors (e.g. age, type of index fracture) that have been identified as increasing the risk of subsequent fracture in other populations [20].

## Methods

### Study participants and design

This study was a retrospective analysis of a German research database containing the claims data of approximately 3 million statutory health insurants from 2007 to 2017. The database is hosted by Vilua Healthcare GmbH (formerly Arvato Health Analytics GmbH) and includes patient characteristics, inpatient and outpatient care diagnoses, drug prescriptions, procedural codes and healthcare cost information. It is representative of Germany in terms of age, sex, morbidity, mortality and geographical distribution [21–24].

Men and women aged 50 years or older who experienced at least one fracture between January 1, 2010 and December 31, 2014 were included in the study. The index fracture was defined as the first fracture experienced by each patient during these 5 years. The study was divided into two distinct time periods, which were calculated individually for each patient: a 3-year baseline period preceding the quarter in which the index fracture occurred, and a 1-year follow-up period following the index fracture quarter. A 1-year follow-up period was chosen, because based on van Geel et al., the risk of subsequent fracture is highest within the first year after initial fracture [14]. Since index fractures had to occur between 2010 and 2014, the earliest the baseline period could begin was January 1, 2007, and the latest the follow-up period could finish was December 31, 2015 (see Supplementary Fig. 1).

Patients were excluded if they had suffered a previous fracture during the year preceding the index fracture quarter; this was to prevent the inclusion of patients that sustained an index fracture during the final year of their baseline period. To limit the analyses to individuals with osteoporosis, included patients must have had a diagnosis of osteoporosis (based on ICD-10-GM code M80.*/M81.*) or a prescription for an osteoporosis medication during baseline. Additional exclusion criteria were a diagnosis of osteodystrophia deformans/Paget syndrome (ICD-10-GM code M88), a calcium homeostasis disorder (ICD-10-GM code E83.5) or a fracture due to malignancy (ICD-10-GM code M90.7).

Fractures were defined by ICD-10-GM codes and comprised vertebral (T08, S12, S22, S32), forearm/wrist/hand (S52, S62), hip/femur (S72) and shoulder/upper arm (S42) fractures. Here, all references to the total number of index or subsequent fractures reflect the sum of all four fracture categories (i.e., all eight diagnostic codes). We then focused on the three most common fracture categories: vertebral (T08, S12, S22, S32), forearm/wrist/hand (S52, S62) and hip/femur (S72) fractures. Major osteoporotic fractures (MOFs), including humerus, forearm, hip and vertebral fracture [10], were also analyzed as a distinct category that included the ICD-10-GM codes S22, S32, S42, S52, and S72.

For inpatient diagnoses, the index fracture date was defined as the date of the diagnosis, whereas for outpatient diagnoses, it was defined as the middle date of the quarter in which the diagnosis was made [22]. To differentiate new from existing fractures, a second fracture at the same site as defined by the ICD-10-GM code was only considered a subsequent fracture if it occurred at least 7 months (for inpatient diagnosis) or at least three quarters (for outpatient diagnosis) after the index fracture. All fractures could be coded in an inpatient or outpatient setting, except for hip/femur fractures, which were always assumed to have required an inpatient diagnosis at incidence.

### Statistical analysis

Descriptive statistics were calculated for patients’ baseline characteristics and the incidence of index and subsequent fractures. Absolute counts and percentages are presented for categorical variables; means and standard deviations (SDs) are presented for continuous variables. Unadjusted odds ratios (ORs) and corresponding 95% confidence intervals for the various index and subsequent fracture types were calculated with chi-squared tests using PSPP version 1.0.1. Index hip/femur fracture was used as the reference group when calculating unadjusted ORs.

Cox proportional hazards models were used to identify variables independently associated with the time interval between the index fracture and any subsequent fracture sustained during the 1-year follow-up period. “Any” subsequent fracture encompassed the ICD-10-GM codes T08, S12, S22, S32, S42, S52, S62 and S72. The list of candidate risk factors, based largely on the Dachverband Osteologie e.V. (DVO) guidelines for osteoporosis [25], was as follows: sex, age, index fracture type, osteoporosis medication, Charlson Comorbidity Index (CCI) score, 17 medical conditions not including osteoporosis (e.g. rheumatoid arthritis [RA], stroke), and eight non-osteoporosis medication categories (e.g. glucocorticoids and fall-inducing medications). Medications were identified by prescription (Anatomical Therapeutic Chemical [ATC] classification) codes and osteoporosis medications were categorized as either bisphosphonates (alendronate, risedronate, ibandronate and zoledronate) or other (raloxifene, denosumab, teriparatide, parathyroid hormone and strontium ranelate). Fall-inducing medications included sedatives and hypnotics, antidepressants, Parkinson’s drugs, diuretics, and anti-hypertensives. A full list of candidate risk factors and their associated diagnostic/ATC codes is presented in Supplementary Table 1.

Variables were selected for further analysis by running a univariate Cox proportional hazards model for each candidate risk factor. All variables that were associated (*p* < 0.1) with subsequent fracture risk in these univariate models were incorporated into a reduced multivariate Cox regression model, which had a statistical significance threshold of *p* < 0.05. Age measured in 10-year age bands was included in all multivariate analysis as a covariate. In addition, separate univariate Cox proportional hazard models were performed to identify variables associated (*p* < 0.01) with the risk of subsequent hip/femur fractures (S72) alone and subsequent vertebral fractures (T08, S12, S22, S32) alone. R (version 3.4.1 for Windows) was used for all regression analyses.

## Results

### Baseline characteristics

A total of 18,354 patients were coded with an index fracture between 2010 and 2014 and were included in the study. This population had a mean age of 77 years (SD = 9.8 years) and was predominantly female (90.2%). Within the study population, 70.8% of patients had an osteoporosis diagnosis prior to index fracture, and the proportion of patients with a diagnosis was greater in females than males (Table 1). Fewer patients overall (34.5%) were prescribed an osteoporosis medication prior to index fracture. The proportion of patients with an osteoporosis medication prescription was greater in females than males (Table 1).

**Table 1:** Baseline characteristics of the study population (*N* = 18,354)

Characteristic (at time of index fracture) n (%), unless otherwise stated	
Mean age, years (SD)	77 (9.8)
Males (SD)	73 (10.6)
Females (SD)	77 (9.6)
Female	16,560 (90.2)
Osteoporosis diagnosis	13,001 (70.8)
Males	1143 (63.7)
Females	11,858 (71.6)
Prescribed osteoporosis medication^a^	6325 (34.5)
Males	537 (29.9)
Females	5788 (35.0)
Comorbidities	
Chronic obstructive pulmonary disease	7337 (40.0)
Congestive heart failure	5936 (32.3)
Dementia	3090 (16.8)
Diabetes w/chronic complication	2495 (13.6)
Hemiplegia/paraplegia	1000 (5.4)
HIV/AIDS	< 10 (0.0)
Malignancy	3336 (18.2)
Metastatic solid tumor	758 (4.1)
Mild liver disease	3289 (17.9)
Moderate/severe liver disease	142 (0.8)
Myocardial infarction	2371 (12.9)
Renal disease	3561 (19.4)
Rheumatic disease	2379 (13.0)
Stroke	1515 (8.3)
Charleston Comorbidity Index (≥ 2)	12.007 (65.4)

^a^Osteoporosis medication defined as bisphosphonates (alendronate, risedronate, ibandronate and zoledronate) or “other medication”: Non-BiP OP medication, BiP prescription possible (raloxifene, denosumab, teriparatide, parathyroid hormone and strontium ranelate)

*SD* standard deviation

Vertebral fractures were the most common index fracture type in both males (64.9%) and females (48.1%), whereas hip/femur fractures were the least common (males: 12.8%; females: 15.0%) (Table 2). 85.2% of all index fractures were MOFs (Table 2). In patients with index vertebral, hip/femur and forearm/wrist/hand fractures specifically, the proportion of patients with a prior diagnosis of osteoporosis was 72.2% (*n* = 6591), 70.1% (*n* = 1897) and 68.6% (*n* = 3055), respectively. The proportion of patients previously prescribed an osteoporosis medication was greater for index vertebral fractures (*n* = 3577; 39.2%) than for index forearm/wrist/hand (*n* = 1387; 31.1%) or hip/femur fractures (*n* = 773; 28.6%).

**Table 2:** Summary of the study population by age and index fracture type (*N* = 18,354)

Patient characteristics			Type of index fracture, *n* (%)^a^			
Sex	Age group (years)	*n*	Hip/femur	Vertebral	Forearm/wrist/hand	MOF
Females	50–59	828	33 (4.0)	395 (47.7)	317 (38.3)	645 (77.9)
	60–69	2049	128 (6.2)	903 (44.1)	758 (37.0)	1693 (82.6)
	70–79	5806	593 (10.2)	2945 (50.7)	1582 (27.2)	4850 (83.5)
	80–89	6276	1241 (19.8)	3020 (48.1)	1294 (20.6)	5524 (88.0)
	≥ 90	1601	481 (30.0)	705 (44.0)	240 (15.0)	1473 (92.0)
	All	16,560	2476 (15.0)	7968 (48.1)	4191 (25.3)	14,185 (85.7)
Males	50–59	235	13 (5.5)	164 (69.8)	42 (17.9)	174 (74.0)
	60–69	359	32 (8.9)	240 (66.9)	58 (16.2)	280 (78.0)
	70–79	643	67 (10.4)	439 (68.3)	94 (14.6)	517 (80.4)
	80–89	472	102 (21.6)	273 (57.8)	58 (12.3)	410 (86.9)
	≥ 90	85	15 (17.6)	49 (57.6)	12 (14.1)	73 (85.9)
	All	1794	229 (12.8)	1165 (64.9)	264 (14.7)	1454 (81.0)
Total^b^			2705 (14.7)	9133 (49.8)	4455 (24.3)	15,639 (85.2)

^a^Hip/femur fracture includes ICD-10-GM [International Classification of Diseases, 10th revision, German Modification] code S72; vertebral fracture includes T08, S12, S22 and S32; forearm/wrist/hand fracture includes S52 and S62; MOF includes S22, S32, S42, S52 and S72

^b^Percentages are relative to the total number of fractures (*N* = 18,354), which includes ICD-10-GM codes T08, S12, S22, S32, S42, S52, S62 and S72. Percentages do not sum to 100 because the fracture categories are not mutually exclusive

*MOF* major osteoporotic fracture

### Patterns of subsequent fractures

**Fig. 1:**
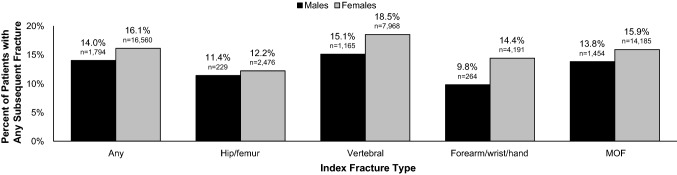
Subsequent fracture incidence by index fracture type and sex (*N* = 18,354). Any fracture encompasses ICD-10-GM [International Classification of Diseases, 10th revision, German Modification] codes T08, S12, S22, S32, S42, S52, S62 and S72. Hip/femur fracture includes ICD-10-GM code S72; vertebral fracture includes T08, S12, S22 and S32; forearm/wrist/hand fracture includes S52 and S62; MOF includes S22, S32, S42, S52 and S72. The n values represent the number of patients that suffered a specific index fracture, while the percentages reflect the proportion of these patients that went on to sustain any subsequent fracture. *MOF* major osteoporotic fracture

Among index fracture patients, 2918 (15.9%) were coded with a subsequent fracture and 1703 (9.3%) died during the 1-year follow-up period. Among patients with a coded subsequent fracture within 1 year, the mean time elapsed between the index and subsequent fracture was 223 days. Incidence of subsequent fracture was greatest amongst patients with an index vertebral fracture (*n* = 1647; 18.0%), followed by those with an index forearm/wrist/hand (*n* = 628; 14.1%) or hip/femur fracture (*n* = 327; 12.1%). Amongst patients with an index MOF, 15.7% (*n* = 2450) went on to suffer a subsequent fracture; 2073 (84.6%) of these were also MOFs. A greater proportion of females (*n* = 2666; 16.1%) than males (*n* = 252; 14.0%) had a diagnostic code for a subsequent fracture (Fig. 1). The difference in subsequent fracture incidence by sex was greatest in patients with index forearm/wrist/hand fractures (14.4% in females vs. 9.8% in males).

**Fig. 2:**
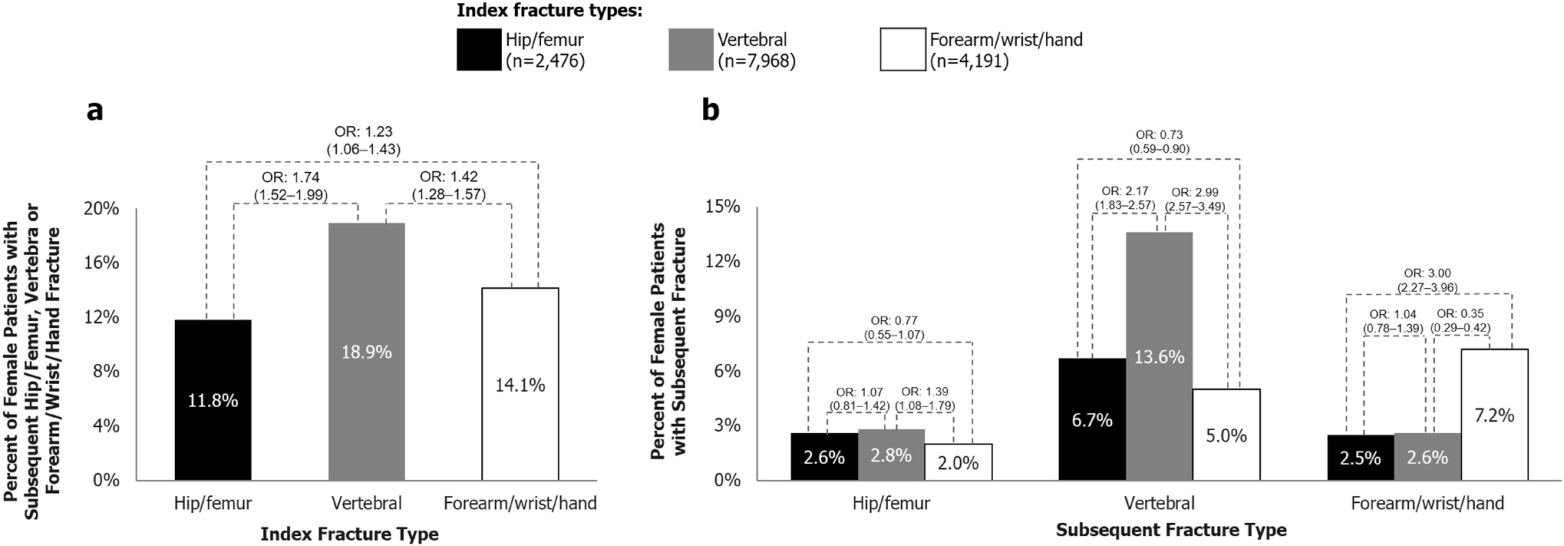
Patterns of **a** subsequent hip/femur, vertebral or forearm/wrist/hand fracture and **b** each type of subsequent fracture in females (*N* = 14,635). Hip/femur fracture includes ICD-10-GM [International Classification of Diseases, 10th revision, German Modification] code S72; vertebral fracture includes T08, S12, S22 and S32; forearm/wrist/hand fracture includes S52 and S62. Index hip/femur fractures comprise the reference group for ORs. *OR* odds ratio (95% confidence interval). Odds ratios are unadjusted

**Fig. 3:**
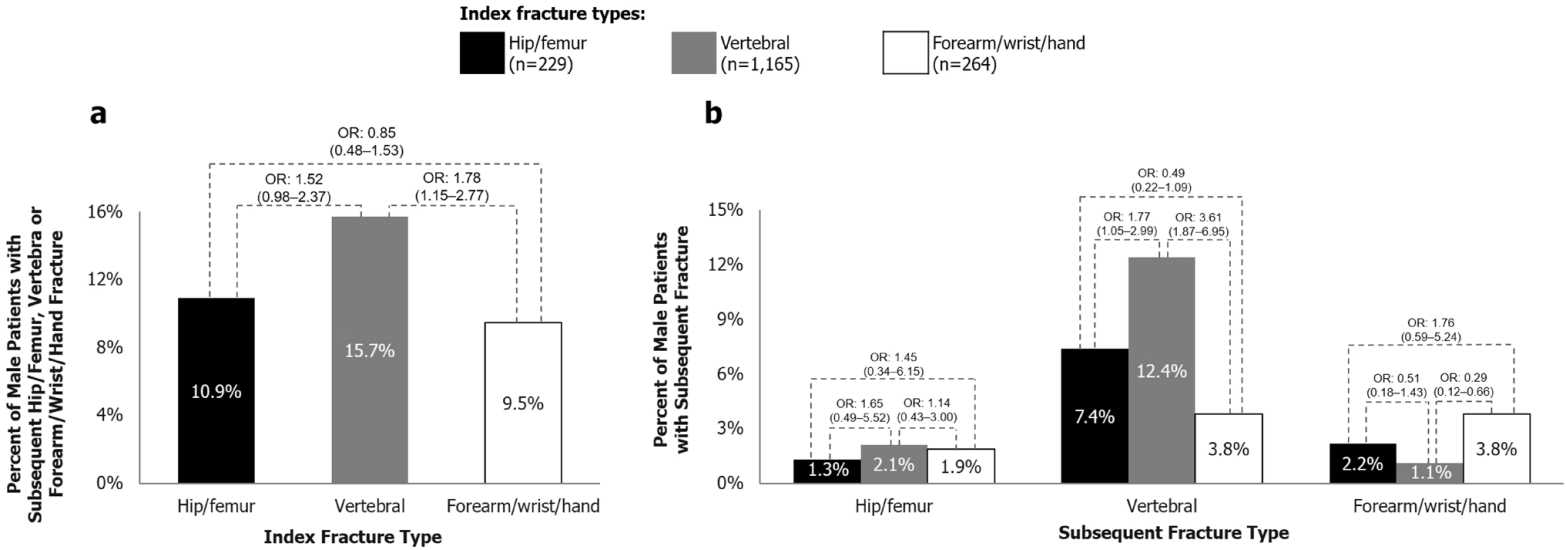
Patterns of **a** subsequent hip/femur, vertebral or forearm/wrist/hand fracture and **b** each type of subsequent fracture in males (*N* = 1658). Hip/femur fracture includes ICD-10-GM [International Classification of Diseases, 10th revision, German Modification] code S72; vertebral fracture includes T08, S12, S22 and S32; forearm/wrist/hand fracture includes S52 and S62. Index hip/femur fractures comprise the reference group for ORs. *OR* odds ratio (95% confidence interval). Odds ratios are unadjusted

Descriptive data concerning the relationship between index fracture type and subsequent fracture type are depicted in Figs. 2 and 3. Amongst female patients with an index vertebral fracture, 13.6% suffered a subsequent vertebral fracture, but only 2.8% and 2.6% suffered a subsequent hip/femur or forearm/wrist/hand fracture, respectively (Fig. 2b). Amongst female index hip/femur fracture patients, 6.7% sustained a subsequent vertebral fracture, but only 2.6% and 2.5% sustained a subsequent hip/femur or forearm/wrist/hand fracture, respectively (Fig. 2b). In female patients with an index forearm/wrist/hand fracture, 7.2% and 5.0% sustained a subsequent forearm/wrist/hand or vertebral fracture, respectively, but only 2.0% suffered a subsequent hip/femur fracture (Fig. 2b). Similar patterns were observed in male patients (Fig. 3b).

The likelihood of subsequent fracture was generally higher in older patients; this was the case for all index fracture types except index hip/femur fractures, for which the incidence of subsequent fractures decreased with increasing age (Supplementary Table 2).

### Risk factors for subsequent fracture

Candidate risk factors for any subsequent fracture during follow-up were analyzed via regression modeling (Table 3). A total of 13 variables were included in the final multivariate model: index fracture type, sex, age, osteoporosis medication (categorized as bisphosphonates or other), antidepressants, diuretics, fall-inducing medications, proton pump inhibitors, CCI score, epilepsy/use of antiepileptics, heart failure, hypoosmolality and hyponatremia, and RA. Index fracture type and age were both found to be significantly associated with subsequent fracture risk when controlling for other variables. Relative to patients with index vertebral fractures, those with index hip/femur (hazard ratio [HR]: 0.65; *p* < 0.001) and forearm/wrist/hand fractures (HR: 0.76; *p* < 0.001) were at lower risk of subsequent fracture. Risk increased with age; relative to patients aged 70–79 years, those aged 50–59 (HR: 0.74; *p* = 0.001) and 60–69 (HR: 0.83; *p* = 0.003) were at lower risk. Epilepsy/use of antiepileptics (HR: 1.13; *p* = 0.017) and heart failure (HR: 1.12; *p* = 0.021) were also independently associated with subsequent fracture risk, but sex, RA, CCI score, osteoporosis medication and all other medications were not (Table 3). Additional univariate analyses revealed that age, index fracture type and osteoporosis medication were each associated with the risk of subsequent hip/femur fractures alone and subsequent vertebral fractures alone (Supplementary Table 3).

**Table 3:** Risk factors that predict the time interval between an index fracture and any subsequent fracture

Variable	Univariate analyses (any subsequent fracture)^a^		Multivariate analysis (any subsequent fracture)^a^	
	Hazard ratio (95% CI)	*p* value^b^	Hazard ratio (95% CI)	*p* value^b,c^
Index fracture type (reference group: index vertebral fracture [*n* = 9133])^a^				
Index fracture: Hip/femur (*n* = 2705)	0.69 (0.61, 0.77)	< 0.001	**0.65 (0.58, 0.74)**	**< 0.001**
Index fracture: Forearm/wrist/hand (*n* = 4455)	0.74 (0.67, 0.81)	< 0.001	**0.76 (0.70, 0.84)**	**< 0.001**
Index fracture: Shoulder/upper arm (*n* = 2061)	0.83 (0.74, 0.94)	0.003	**0.84 (0.75, 0.95)**	**0.006**
Sex (reference group: males [*n* = 1794])				
Females [*n* = 16,560]	1.13 (0.99, 1.29)	0.065	1.12 (0.98, 1.28)	0.087
Age group (reference group: 70–79 years [*n* = 6449])				
50–59 years (*n* = 1063)	0.71 (0.59, 0.85)	< 0.001	**0.74 (0.61, 0.88)**	**0.001**
60–69 years (*n* = 2408)	0.80 (0.71, 0.91)	< 0.001	**0.83 (0.73, 0.94)**	**0.003**
80–89 years (*n* = 6748)	1.05 (0.96, 1.14)	0.271	1.05 (0.96, 1.14)	0.280
≥ 90 years (*n* = 1686)	1.14 (1.00, 1.30)	0.048	**1.17 (1.02, 1.34)**	**0.026**
Osteoporosis medication (reference group: no osteoporosis medication [*n* = 12,029])				
Bisphosphonates only (*n* = 5828)	1.10 (1.02, 1.19)	0.020	1.05 (0.97, 1.14)	0.206
Other osteoporosis medication (*n* = 885)	1.10 (0.93, 1.30)	0.259	1.05 (0.89, 1.24)	0.560
Other medications (reference group: no other medications)				
Antidepressants^d^ (*n* = 5975)	1.13 (1.05, 1.22)	0.002	1.07 (0.99, 1.16)	0.079
Aromatase inhibitors (*n* = 410)	0.95 (0.74, 1.23)	0.706	–	–
Diuretics^d^ (*n* = 8017)	1.09 (1.02, 1.18)	0.017	0.94 (0.86, 1.02)	0.134
Glucocorticoids (4619)	1.01 (0.93, 1.10)	0.728	–	–
Hormonablative therapy/antiandrogen therapy in males (*n* = 36)	0.33 (0.08, 1.32)	0.117	–	–
Fall-inducing medications(*n* = 3794)	1.16 (1.06, 1.26)	0.001	1.07 (0.98, 1.18)	0.120
Proton pump inhibitor (PPI) (*n* = 10,632)	1.12 (1.04, 1.21)	0.002	1.04 (0.97, 1.13)	0.276
Thiazolidindiones (glitazones) in females (*n* = 79)	1.22 (0.73, 2.02)	0.452	–	–
Charlson Comorbidity Index score (reference group: Charlson Comorbidity Index score ≤ 1 [***n*** = 6347])				
Charlson Comorbidity Index score > 1 (*n* = 12.007)	1.18 (1.10, 1.28)	< 0.001	1.06 (0.97, 1.16)	0.181
Medical conditions (reference group: no condition)				
Stroke (*n* = 1515)	1.03 (0.91, 1.18)	0.630	–	–
Myocardial infarction (*n* = 2371)	1.09 (0.98, 1.21)	0.100	–	–
Ankylosing spondylitis (*n* = 145)	1.25 (0.87, 1.80)	0.231	–	–
Chronic obstructive pulmonary disease (*n* = 7337)	1.01 (0.92, 1.11)	0.836	–	–
Subclinical hypercortisolism and Cushing’s syndrome (*n* = 48)	0.96 (0.46, 2.03)	0.925	–	–
Diabetes mellitus type 1 (*n* = 977)	1.06 (0.90, 1.24)	0.496	–	–
Diabetes mellitus type 2 (*n* = 4744)	1.00 (0.92, 1.08)	0.920	–	–
Epilepsy/use of antiepileptics (*n* = 2569)	1.19 (1.08, 1.32)	0.001	**1.13 (1.02, 1.26)**	**0.017**
Growth hormone deficiency (*n* = 32)	0.76 (0.28, 2.02)	0.576	–	–
Heart failure (*n* = 5379)	1.23 (1.13, 1.32)	< 0.001	**1.12 (1.02, 1.23)**	**0.021**
Other specialized nutritional anemias (*n* = 6)	2.42 (0.60, 9.67)	0.212	–	–
Hypoosmolality and hyponatremia (*n* = 897)	1.17 (0.99, 1.37)	0.060	1.06 (0.89, 1.24)	0.522
Monoclonal gammopathy of unclear significance (*n* = 132)	0.94 (0.60, 1.48)	0.793	–	–
Primary hyperparathyroidism (*n* = 43)	1.13 (0.56, 2.26)	0.730	–	–
Rheumatoid arthritis (*n* = 1941)	1.13 (1.01, 1.26)	0.036	1.10 (0.98, 1.23)	0.112
Subclinical and manifest hyperthyreosis (*n* = 1881)	1.04 (0.93, 1.17)	0.497	–	–
Vitamin D and calcium deficiency (*n* = 717)	0.88 (0.72, 1.07)	0.207	–	–

*CI* confidence interval

^a^Any fracture encompasses ICD-10-GM [International Classification of Diseases, 10th revision, German Modification] codes T08, S12, S22, S32, S42, S52, S62 and S72. Hip/femur fracture includes ICD-10-GM code S72; vertebral fracture includes T08, S12, S22 and S32; forearm/wrist/hand fracture includes S52 and S62; shoulder/upper arm fracture includes S42

^b^Grey values are significant at the 95% level following a univariate or multivariate regression analysis as listed. Variables that were associated with subsequent fracture risk following univariate analysis (*p* < 0.1) were incorporated into multivariate regression analysis

^c^Bold values were identified as independently associated with fracture risk after multivariate regression

^d^These medications also lead to an increased inclination for falls

## Discussion

This is one of the first studies to characterize subsequent fracture risk following an index fracture in individuals with osteoporosis in Germany. Of the 18,354 osteoporotic patients in this study that had a coded index fracture between 2010 and 2014, around one in six went on to sustain a subsequent fracture during the 1-year follow-up period.

The concept of imminent risk following an initial osteoporotic fracture is well established, based on analyses of patient data from North America, Europe, East Asia and Australasia [13, 17, 20, 26, 27]. Our results indicate that imminent risk is similarly present in the German population. Here, 15.9% of osteoporosis patients with a history of at least one fracture suffered a subsequent fracture during the 1-year follow-up period, which is substantially higher than fracture rates reported in samples of osteoporotic patients not limited to individuals with a prior fracture. For instance, an earlier German study found that 10.7% of male osteoporosis patients aged ≥ 60 years and 9.5% of female osteoporosis patients aged ≥ 55 years sustained new vertebral or hip/femur fractures in 2016 [2]. Our results also suggest that the imminent risk of subsequent fracture is somewhat higher in Germany than in other countries, although this may reflect differences in the patient demographics and/or fracture coding practices used in these studies [11, 14, 19, 20, 28].

The annual incidence of osteoporosis-associated femoral fractures in females aged 75–79 years in Germany is approximately 0.6% [29]. In this study, the 1-year cumulative incidence of *subsequent* hip/femur fracture was between 2.0 and 2.8% for female osteoporosis patients with an average age of 77. Hence, index fracture is associated with a considerable increase in hip/femur fracture risk.

Subsequent fractures result in poor health, social, and economic outcomes; a subsequent hip fracture, for example, is associated with decreased mobility and social independence, as well as increased mortality [28, 30–32]. The heightened fracture risk within the 1–2 years following an index fracture highlights the need for immediate treatment after an index fracture. In Germany, however, around 60% of women aged ≥ 50 years remain untreated during the first year after an osteoporotic fracture [10], highlighting a missed opportunity to treat patients at imminent risk of fracture.

The incidence of subsequent fractures was greatest in older patients, echoing earlier findings that each year of life increases subsequent MOF risk [17]. Patients sustaining an index vertebral fracture were at elevated risk of subsequent fractures compared to patients with other index fracture types. This finding is consistent with evidence suggesting that vertebral fractures often precede additional fractures as part of a “fracture cascade” [16].

The number of men and women were imbalanced as typically found in osteoporosis populations. As the multivariate analysis did not comprise sex as an independent risk factor, we believe that excluding men would not have a significant impact on the overall results.

Index fracture type, age and sex were all identified as risk factors for subsequent fracture in our univariate analyses. However, the female patient group was on average older than the male group (77 vs. 73 years, respectively), and sex was not significantly associated with subsequent fracture risk when considered as part of our final multivariate model. In contrast, index fracture type, age, epilepsy/use of antiepileptics and heart failure were all found to be independently associated with subsequent fracture risk. These risk factors are already featured in the DVO guidelines as variables that are predictive for osteoporotic fractures [25], and our latest results confirm their impact on subsequent fracture risk [33]. Therefore, it is likely that epilepsy/use of antiepileptics contribute to an imminent risk of fracture by an increased risk of falls. In contrast, fall-inducing medications were not identified as an independent risk factor for imminent risk of fracture in our multivariate analysis (Table 3).

Treatment of such as use of Bisphosphonates were not associated with lower risk for a subsequent fracture. In our database, we did not have access to the exact date of treatment initiation for all of the patients. Based on previous reports in Germany, we assume that, only a minority of patients receive a treatment directly after an osteoporosis-related fracture, [34] which would explain the small impact of treatment on subsequent fracture risk. In addition, the small impact of osteoporosis treatments on subsequent fracture risk could also be explained by the documented low persistence to treatment. As previously shown, in several German-based studies, persistency with oral bisphosphonates, the first-line treatment in Germany, were reported to be as low as 20% after 12 months of follow-up [35–37].

Previous evidence suggests that numerous other variables are associated with osteoporotic fractures, including RA, type I diabetes, chronic obstructive pulmonary disease, Cushing’s syndrome, androgen therapy, glucocorticoids, antidepressants and aromatase inhibitors [25], yet none were identified as independent risk factors for subsequent fracture in the present study. Glucocorticoid-induced osteoporosis is known to increase patients’ fracture risk [25, 28, 38–40], but the association between glucocorticoid use and subsequent fracture risk did not approach significance (*p* = 0.728) in our univariate analysis. In addition, RA confers heightened fracture risk independently of glucocorticoid use [25], yet RA was not significantly associated with subsequent fracture risk in our multivariate model. It is conceivable that the key risk factors for subsequent fractures differ from the established list of osteoporotic fracture risk factors, and/or that the importance of specific risk factors is dependent on timing within the baseline period. However, glucocorticoid use, fall-inducing medications, rheumatic disease and CCI were all identified as independent risk factors in a recent Swedish study of subsequent fracture risk [20]. Again, different fracture coding practices could account for these findings.

This study had several limitations. First, data for several established risk factors were not available for inclusion in our regression analyses, including bone mineral density, body mass index, smoking, fall history, total number of previous fractures and parental history of hip fracture. Second, while imminent risk was assessed in a large cohort of osteoporotic patients sampled from the German general population, the *increase* in fracture risk associated with a prior fracture could only be estimated through comparison with incidence rates from the published literature. Third, previous research has demonstrated that subsequent fracture risk is highest within the 1–2 years following an initial osteoporotic fracture [14, 17, 18], but in the present study, subsequent fracture risk was only assessed over a 1-year follow-up period. Consequently, subsequent fractures that occurred after this point were not captured in our dataset, limiting the power and scope of the study and its conclusions. Furthermore, the average time interval between index and subsequent fractures was limited by the 1-year duration of the follow-up period; as such, it is not anticipated to reflect the average fracture interval in the wider osteoporotic population. In addition, while distal forearm/wrist fracture as well as fractures of the humerus are typical osteoporotic fracture and also contribute to the count as major osteoporotic fractures, all patients also had an OP diagnosis or a prescription for OP medication. These, for OP patients, rare fractures of shoulder and were included for reasons of completeness. However, since the absolute and relative number of hand and shoulder is very low, we are confident that the results are not affected by this broader definition.

In our analyses, patients were categorized according to three- rather than four-character ICD-10-GM codes. While three-character codes have been used in other real-world analyses of osteoporotic fracture frequency in Germany [34, 41], four-character ICD-10-GM codes offer greater specificity; in particular, they allow the differentiation of clinical and non-clinical fractures. It is, therefore, possible that some non-clinical vertebral fractures were captured here. Consistent with this suggestion, the ratio of vertebral-to-hip/femur index fractures in our dataset was considerably higher than documented elsewhere [42], although other studies have reported a substantially greater number of vertebral than hip/femur index fractures [43–45]. The use of three-character ICD-10-GM codes may also have resulted in the inclusion of some non-osteoporotic fractures, although all patients were diagnosed with osteoporosis and/or prescribed osteoporosis medication prior to index fracture, so it is likely that most were osteoporosis-related. Lastly, the algorithms we used to identify incident fractures are not yet validated; some subsequent fractures may actually have been existing fractures recorded at follow-up visits, resulting in an overestimation of subsequent fracture incidence.

## Conclusions

Osteoporosis patients in Germany sustaining an initial fracture are at imminent risk of subsequent fracture, with approximately one in six patients with a coded subsequent fracture during the 1-year follow-up period. Immediate treatment following an index fracture could prevent subsequent fractures, reducing the associated negative health, social, and economic consequences. Clinicians should consider the recency of patients’ previous fracture(s) when making treatment decisions, paying particular attention to older adults and patients with comorbidities that may put them at increased risk of subsequent fracture.

## Supplementary Information

Below is the link to the electronic supplementary material.

Supplementary file1 (DOCX 119 KB)
