# Supplementary material for: Osteoporotic fractures and subsequent fractures: imminent fracture risk from an analysis of German real-world claims data
Source: Arch Gynecol Obstet. 2021 Jul 11;304(3):703–12. doi: 10.1007/s00404-021-06123-6 (PMC8325652; doi:10.1007/s00404-021-06123-6)
Supplement: Supplementary file 1 — Supplementary file1 (DOCX 119 KB) [file 404_2021_6123_MOESM1_ESM.docx]

SUPPLEMENTARY DATA

Supplementary Fig. 1 Study design


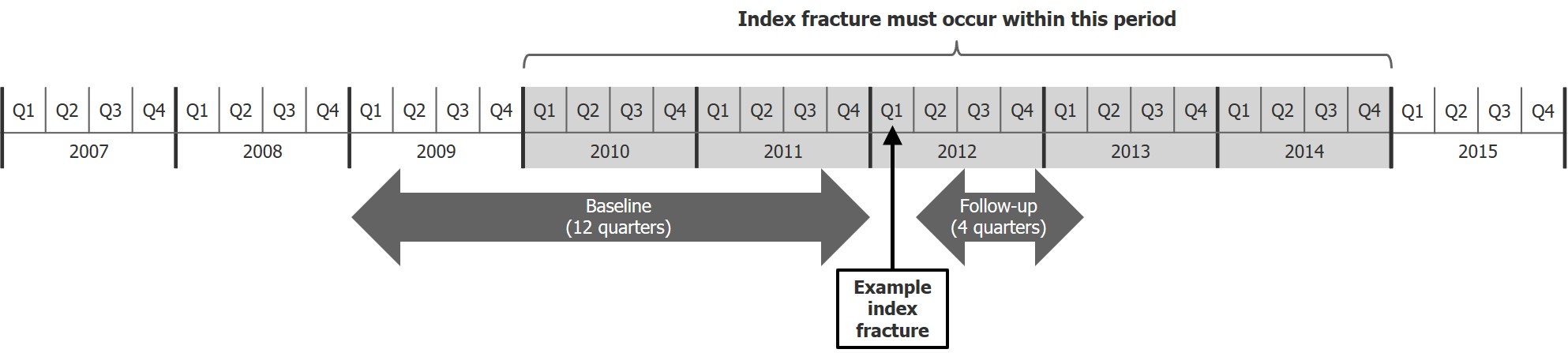


Q: quarter.

**Supplementary Table 1** Candidate risk factors for subsequent fracture included in the Cox proportional hazards models

| **Variable** | **Description, diagnostic and/or prescription code(s)** |
| --- | --- |
| Age | 50–59, 60–69, 70–79, 80–89 or ≥90 years |
| Sex | Male or female |
| Index fracture type | ICD S72 (hip/femur fracture); ICD T08, S12, S22, S32 (vertebral fracture); ICD S52, S62 (forearm/wrist/hand fracture); ICD S42 (shoulder/upper arm fracture); ICD S22, S32, S42, S52, S72 (MOF) |
| Charlson Comorbidity Index (CCI) | Calculated following the updated methodology suggested by Quan et al. [[46](#_ENREF_46)] |
| **Osteoporosis medication** | |
| Bisphosphonates only | ATC M05BA04 (alendronate), ATC M05BA07 (risedronate), ATC M05BA06 (ibandronate), ATC M05BA08 (zoledronate) |
| Other osteoporosis medication | ATC G03XC01 (raloxifene), ATC M05BX04 (denosumab), ATC H05AA02 (teriparatide), ATC H05AA03 (parathyroid hormone), ATC M05BX03 (strontium ranelate) |
| **Non-osteoporosis medication** | |
| Antidepressants^a^ | ATC N064 |
| Aromatase inhibitors | ATC L02BG03 (anastrozol), ATC L02BG04 (letrozol), ATC L02BG06 (exemestan) |
| Diuretics^a^ | ATC C03 |
| Glucocorticoids | ATC H02AB03 (fluocortolone), ATC H02AB04 (methylprednisolone), ATC H02AB06 (prednisolone), ATC H02AB07 (prednisone), ATC H02AB08 (triamcinolone), ATC H02AB09 (hydrocortisone), ATC H02AB10 (cortisone), ATC H02AB11 (prednylidene), ATC H02AB13 (deflazacort) |
| Hormonablative therapy/antiandrogen therapy in males | ATC G03BA (3-oxoandrosten-(4) derivatives), ATC G03BB (5-androstanon-(3) derivatives) |
| Fall-inducing medications | ATC N05C (sedatives and hypnotics), ATC N06A (antidepressants), ATC N04 (anti-Parkinson’s), C03 (diuretics), ATC C02 (anti-hypertensives) |
| Proton pump inhibitor (PPI) | ATC A02BC |
| Thiazolidindiones (glitazones) in females | ATC A10BG |
| **Non-osteoporosis medical conditions** | |
| Stroke | ICD I60.*, ICD I61.*, ICD I62.*, ICD I63.*, ICD I64.* |
| Myocardial infarction | ICD I20.*, ICD I21.*, ICD I22.* |
| Ankylosing spondylitis | ICD M45 |
| Chronic obstructive pulmonary disease | ICD J44 |
| Subclinical hypercortisolism and Cushing’s syndrome | ICD E24 |
| Diabetes mellitus type 1 | ICD E10 |
| Diabetes mellitus type 2 | ICD E11 |
| Epilepsy/use of antiepileptics | ICD G40 (epilepsy), ATC N03 (antiepileptic medication) |
| Growth hormone deficiency | ICD E23.0 |
| Heart failure | ICD I50 |
| Other specialized nutritional anemias | ICD D53.8 |
| Hypoosmolality and hyponatremia | ICD E87.1 |
| Monoclonal gammopathy of unclear significance | ICD D47.2 |
| Primary hyperparathyroidism | ICD E21.0 |
| Rheumatoid arthritis | ICD M05 (rheumatoid arthritis with rheumatoid factor), ICD M06 (other rheumatoid arthritis) |
| Subclinical and manifest hyperthyreosis | ICD E05 |
| Vitamin D and calcium deficiency | ICD E55 (vitamin D deficiency), ICD E58 (calcium deficiency) |

^a^These medications also lead to an increased inclination for falls. ATC: Anatomical Therapeutic Chemical Classification; ICD: International Classification of Diseases; N/A: not applicable.

**Supplementary Table 2** Subsequent fracture incidence by index fracture type, sex and age (N=18,354)

| **Index fracture type^a^** | **Sex** | **Age group (years)** | **n** | **With any subsequent fracture, n (%)^a^** |
| --- | --- | --- | --- | --- |
| Hip/femur | Females | 50–59 | 33 | 2 (6.1) |
|  |  | 60–69 | 128 | 20 (15.6) |
|  |  | 70–79 | 593 | 78 (13.2) |
|  |  | 80–89 | 1241 | 148 (11.9) |
|  |  | ≥90 | 481 | 53 (11.0) |
|  | Males | 50–59 | 13 | 2 (15.4) |
|  |  | 60–69 | 32 | 4 (12.5) |
|  |  | 70–79 | 67 | 8 (11.9) |
|  |  | 80–89 | 102 | 12 (11.8) |
|  |  | ≥90 | 15 | 0 |
| Vertebral | Females | 50–59 | 395 | 64 (16.2) |
|  |  | 60–69 | 903 | 132 (14.6) |
|  |  | 70–79 | 2945 | 549 (18.6) |
|  |  | 80–89 | 3020 | 595 (19.7) |
|  |  | ≥90 | 705 | 131 (18.6) |
|  | Males | 50–59 | 164 | 13 (7.9) |
|  |  | 60–69 | 240 | 37 (15.4) |
|  |  | 70–79 | 439 | 74 (16.9) |
|  |  | 80–89 | 273 | 40 (14.7) |
|  |  | ≥90 | 49 | 12 (24.5) |
| Forearm/wrist/hand | Females | 50–59 | 317 | 33 (10.4) |
|  |  | 60–69 | 758 | 94 (12.4) |
|  |  | 70–79 | 1582 | 234 (14.8) |
|  |  | 80–89 | 1294 | 201 (15.5) |
|  |  | ≥90 | 240 | 40 (16.7) |
|  | Males | 50–59 | 42 | 4 (9.5) |
|  |  | 60–69 | 58 | 9 (15.5) |
|  |  | 70–79 | 94 | 7 (7.4) |
|  |  | 80–89 | 58 | 3 (5.2) |
|  |  | ≥90 | 12 | 3 (25.0) |
| MOF | Females | 50–59 | 645 | 90 (14.0) |
|  |  | 60–69 | 1693 | 220 (13.0) |
|  |  | 70–79 | 4850 | 811 (16.7) |
|  |  | 80–89 | 5524 | 896 (16.2) |
|  |  | ≥90 | 1473 | 232 (15.8) |
|  | Males | 50–59 | 174 | 15 (8.6) |
|  |  | 60–69 | 280 | 39 (13.9) |
|  |  | 70–79 | 517 | 74 (14.3) |
|  |  | 80–89 | 410 | 57 (13.9) |
|  |  | ≥90 | 73 | 16 (21.9) |

^a^Any fracture encompasses ICD-10-GM [International Classification of Diseases, 10th revision, German Modification] codes T08, S12, S22, S32, S42, S52, S62 and S72. Hip/femur fracture includes ICD-10-GM code S72; vertebral fracture includes T08, S12, S22 and S32; forearm/wrist/hand fracture includes S52 and S62; MOF includes S22, S32, S42, S52 and S72. MOF: major osteoporotic fracture.

**Supplementary Table 3** Risk factors that predict the time interval between an index fracture and a subsequent hip/femur fracture or a subsequent vertebral fracture

|  | **Univariate analyses**  **(Subsequent hip/femur fracture)^a^** | | **Univariate analyses**  **(Subsequent vertebral fracture)^a^** | |
| --- | --- | --- | --- | --- |
| **Variable** | **Hazard ratio (95% CI)** | **p value^b^** | **Hazard ratio (95% CI)** | **p value^b^** |
| **Index fracture type (reference group: index vertebral fracture)^a^** | | | | |
| Index fracture: Hip/femur | 0.92 (0.69, 1.23) | 0.559 | 0.54 (0.46, 0.63) | <0.001 |
| Index fracture: Forearm/wrist/hand | 0.69 (0.53, 0.90) | 0.005 | 0.34 (0.30, 0.40) | <0.001 |
| Index fracture: Shoulder/upper arm | 0.90 (0.65, 1.24) | 0.507 | 0.46 (0.38, 0.55) | <0.001 |
| **Sex (reference group: males)** | | | | |
| Females | 0.93 (0.83, 1.05) | 0.240 | 1.00 (0.89, 1.13) | 0.984 |
| **Age group (reference group: 70–79 years)** | | | |  |
| 50–59 years | 0.25 (0.09, 0.67) | 0.006 | 0.67 (0.53, 0.85) | 0.001 |
| 60–69 years | 0.44 (0.26, 0.75) | 0.003 | 0.70 (0.59, 0.82) | <0.001 |
| 80–89 years | 2.17 (1.70, 2.76) | <0.001 | 0.94 (0.84, 1.04) | 0.239 |
| ≥90 years | 3.79 (2.82, 5.10) | <0.001 | 0.91 (0.76, 1.09) | 0.309 |
| **Osteoporosis medication (reference group: no osteoporosis medication)** | | | | |
| Bisphosphonates only | 0.78 (0.62, 0.97) | 0.029 | 1.19 (1.08, 1.32) | 0.001 |
| Other osteoporosis medication | 0.80 (0.49, 1.31) | 0.384 | 1.33 (1.09, 1.64) | 0.005 |
| **Other medications (reference group: no other medications)** | | | | |
| Antidepressants^c^ | 1.04 (0.96, 1.13) | 0.295 | 1.02 (0.94, 1.10) | 0.703 |
| Aromatase inhibitors | 1.12 (0.88, 1.42) | 0.351 | 1.08 (0.86, 1.37) | 0.502 |
| Diuretics^c^ | 0.97 (0.90, 1.04) | 0.410 | 1.01 (0.94, 1.09) | 0.747 |
| Glucocorticoids | 0.97 (0.89, 1.06) | 0.486 | 1.05 (0.97, 1.14) | 0.248 |
| Hormonablative therapy/antiandrogen therapy in males | 1.38 (0.69, 2.76) | 0.367 | 1.06 (0.48, 2.37) | 0.881 |
| Fall-inducing medications | 0.92 (0.84, 1.01) | 0.087 | 0.97 (0.88, 1.06) | 0.442 |
| Proton pump inhibitor (PPI) | 0.99 (0.92, 1.07) | 0.797 | 0.98 (0.91, 1.05) | 0.521 |
| Thiazolidindiones (glitazones) in females | 1.20 (0.72, 1.99) | 0.483 | 1.23 (0.74, 2.05) | 0.420 |
| **Charlson Comorbidity Index score (reference group: Charlson Comorbidity Index score ≤1)** | | | | |
| Charlson Comorbidity Index score >1 | 1.03 (0.95, 1.11) | 0.473 | 1.01 (0.94, 1.09) | 0.808 |
| **Medical conditions (reference group: no condition)** | | | | |
| Stroke | 1.10 (0.97, 1.25) | 0.133 | 1.05 (0.92, 1.19) | 0.492 |
| Myocardial infarction | 1.03 (0.92, 1.14) | 0.619 | 0.98 (0.88, 1.10) | 0.745 |
| Ankylosing spondylitis | 1.03 (0.69, 1.54) | 0.880 | 0.78 (0.49, 1.24) | 0.287 |
| Chronic obstructive pulmonary disease | 1.02 (0.93, 1.11) | 0.730 | 1.04 (0.95, 1.14) | 0.405 |
| Subclinical hypercortisolism and Cushing’s syndrome | 1.17 (0.61, 2.25) | 0.640 | 0.50 (0.19, 1.34) | 0.171 |
| Diabetes mellitus type 1 | 1.04 (0.88, 1.21) | 0.661 | 0.99 (0.84, 1.17) | 0.939 |
| Diabetes mellitus type 2 | 0.98 (0.90, 1.06) | 0.591 | 1.03 (0.95, 1.12) | 0.464 |
| Epilepsy/use of antiepileptics | 1.08 (0.98, 1.20) | 0.139 | 1.02 (0.92, 1.13) | 0.681 |
| Growth hormone deficiency | 0.96 (0.40, 2.31) | 0.932 | 0.36 (0.09, 1.43) | 0.145 |
| Heart failure | 1.06 (0.98, 1.14) | 0.172 | 1.00 (0.92, 1.08) | 0.910 |
| Other specialized nutritional anemias | 0.00 (0, Inf) | 0.975 | 2.53 (0.63, 10.11) | 0.190 |
| Hypoosmolality and hyponatremia | 0.97 (0.82, 1.15) | 0.748 | 0.96 (0.81, 1.14) | 0.635 |
| Monoclonal gammopathy of unclear significance | 1.03 (0.67, 1.58) | 0.888 | 1.15 (0.77, 1.71) | 0.504 |
| Primary hyperparathyroidism | 0.84 (0.38, 1.87) | 0.668 | 0.90 (0.40, 2.00) | 0.796 |
| Rheumatoid arthritis | 0.94 (0.84, 1.07) | 0.352 | 1.07 (0.96, 1.20) | 0.238 |
| Subclinical and manifest hyperthyreosis | 1.03 (0.91, 1.16) | 0.629 | 1.12 (1.00, 1.26) | 0.055 |
| Vitamin D and calcium deficiency | 0.96 (0.80, 1.17) | 0.705 | 1.18 (0.99, 1.41) | 0.059 |

^a^Hip/femur fracture includes ICD-10-GM [International Classification of Diseases, 10th revision, German Modification] code S72; vertebral fracture includes T08, S12, S22 and S32; forearm/wrist/hand fracture includes S52 and S62; shoulder/upper arm fracture includes S42. ^b^Grey values are significant at the 95% level following a univariate analysis. ^c^These medications also lead to an increased inclination for falls. CI: confidence interval. Inf: infinity.
